# Regional and Socioeconomic Disparities in Frailty Across Tasmania: Evidence From Island Study Linking Ageing and Neurodegenerative Disease

**DOI:** 10.1111/ajag.70144

**Published:** 2026-03-09

**Authors:** Zhexun Lou, Eddy Roccati, Jane E. Alty, Michele L. Callisaya, James C. Vickers, Emily H. Gordon, Ruth E. Hubbard, David D. Ward

**Affiliations:** ^1^ Centre for Health Services Research, Faculty of Health, Medicine and Behavioural Sciences The University of Queensland Woolloongabba Queensland Australia; ^2^ Australian Frailty Network The University of Queensland Woolloongabba Queensland Australia; ^3^ Wicking Dementia Research and Education Centre University of Tasmania Hobart Tasmania Australia; ^4^ Royal Hobart Hospital Hobart Tasmania Australia; ^5^ Tasmanian School of Medicine University of Tasmania Hobart Tasmania Australia; ^6^ Menzies Institute for Medical Research University of Tasmania Hobart Tasmania Australia; ^7^ Peninsula Clinical School, School of Translational Medicine Monash University Frankston Victoria Australia; ^8^ Department of General and Geriatric Medicine Princess Alexandra Hospital Woolloongabba Queensland Australia

**Keywords:** Australia, frailty, geographic mapping, rural health, socioeconomic disparities in health

## Abstract

**Objective:**

Although frailty appears higher in rural and socioeconomically disadvantaged areas, existing evidence often lacks adjustment for possible population confounders. This study examined the independent associations between geographic remoteness and area‐level socioeconomic status with frailty.

**Methods:**

We constructed a 33‐item frailty index using data from 5740 participants of the Island Study Linking Ageing and Neurodegenerative Disease (ISLAND), a web‐based longitudinal cohort of adults aged 50 years and over in Tasmania, Australia. After linking participant postcodes to Modified Monash Model remoteness and Index of Relative Socioeconomic Advantage and Disadvantage, we examined frailty distribution and its associations with geographic and sociodemographic factors using descriptive statistics, spatial mapping and multivariable linear regression models.

**Results:**

The analytical sample mean age was 69.3 years (SD = 8.0) and most were women (72%). Frailty index scores followed a gamma distribution (mean score = 0.16, SD = 0.09), increased with age and were highest in central and western areas of Tasmania. After adjustment for age, gender, education, retirement and migrant status, frailty index scores were significantly higher in rural towns (β = 0.011 [95% confidence interval, CI = 0.005, 0.016]) and remote communities (β = 0.023 [95% CI = 0.009, 0.038]) than regional centres. Similarly, after full adjustment, compared with areas of the highest socioeconomic advantage, frailty was significantly higher in areas of middle (β = 0.013 [95% CI = 0.007, 0.018]) or low (β = 0.024 [95% CI = 0.018, 0.030]) socioeconomic advantage.

**Conclusions:**

The distribution of frailty across Tasmania varied by geographic remoteness and socioeconomic disadvantage. Integrating frailty assessment into regional health planning may support targeted interventions for vulnerable subpopulations, particularly in rural and disadvantaged communities.

## Policy Impact

Frailty is higher in rural and disadvantaged areas. Incorporating frailty screening into health system planning and targeting resources to remote and deprived communities might support equitable ageing and guide service investment where health vulnerability is greatest.

## 1 Introduction

Frailty is a critical indicator of health vulnerability and its measurement improves risk grading and clinical management [1]. However, frailty is not evenly distributed across populations; it is shaped by both socioeconomic and geographic factors. For example, frailty prevalence is often higher in rural communities [2, 3], reflecting disparities in healthcare access, socioeconomic conditions and health‐promoting behaviours [4]. Understanding and mapping frailty across regions and sociodemographic groups are fundamental to identifying high‐risk areas and tailoring intervention strategies to the specific needs of populations.

Frailty is characterised as the deterioration of physiological capacity and function in several organ systems due to ageing, resulting in an increased risk of experiencing falls, disability, hospitalisation, dementia, death and other adverse outcomes [5, 6]. One approach to detecting frailty involves measuring the accumulation of age‐related deficits in individuals and calculating a frailty index [7]. The frailty index is a proxy measure for ageing and mortality, offering a macroscopic view of a person's health status rather than focussing on singular diseases or functional declines [8]. Measuring frailty provides a comprehensive approach to assessing health risks beyond chronological age and allows for personalised clinical interventions [9].

In Tasmania, Australia, the mix of urban and rural settings in close proximity offers a useful setting for better understanding disparities in frailty, with implications for both national and international contexts. Tasmania has the highest mean age of Australian jurisdictions at almost 43 years, with 21% of its population aged 65 years and above [10]. It also ranks low in socioeconomic standing, with the lowest median weekly earnings [11] and only 53% of students completing high school compared to 76% nationally [12]. While Australia is a high‐income nation with relatively high health expenditure [13, 14], Tasmania experiences disproportionately poor health outcomes, a disparity that highlights unmet opportunities to translate resources into equitable population health gains. In Hobart, Tasmania's capital, over 12% of the population is expected to be frail or pre‐frail in the coming years [15, 16]. However, the burden is expected to be greater in outer regions, where a higher proportion of the population resides [15, 16]. Such trends are not unique to Tasmania [15] and highlight the need for targeted healthcare services and health‐promotion strategies to address the unique health challenges faced by regional populations.

While frailty is known to be elevated in rural and socioeconomically deprived populations [4, 17], the independent contributions of geographic remoteness and socioeconomic disadvantage to frailty remain poorly understood. The Island Study Linking Aging and Neurodegenerative Disease (ISLAND) is an on‐going prospective cohort study in Tasmania investigating health interventions for reducing the risk of developing dementia. Using ISLAND data and the deficit accumulation approach [18], we aimed to measure frailty and examine its spatial and sociodemographic distribution across Tasmania. To achieve this, we pursued three objectives: (1) develop and validate a frailty index for ISLAND; (2) map frailty patterns across geographic regions and sociodemographic status; and (3) evaluate how regionality and socioeconomic disadvantage independently contribute to frailty, after accounting for potentially confounding individual‐level characteristics.

## 2 Methods

### 2.1 Data Source

The Island Study Linking Aging and Neurodegenerative Disease is a web‐based cohort study in Tasmania, Australia, designed to track dementia risk‐related behaviours in adults aged 50 years and above. Participants were recruited through online registration, where they provided basic details (e.g., name, postcode, and email) and consented to be contacted. Eligible individuals completed a series of baseline surveys and formally enrolled in the cohort. Ongoing data collection includes repeated follow‐up surveys, online cognitive assessments and periodic blood sample collection for biomarker analysis. The ISLAND project was approved by the University of Tasmania Human Research Ethics Committee (HREC; reference 18264). The full study protocol is available elsewhere [19].

Data included demographics, health information, cognitive function, dementia risk knowledge and behaviours, motivations for lifestyle and behaviour change, social networks, mental health and quality of life. Blood‐based biomarkers were collected to track biological changes associated with dementia risk every 2 years [20]. Of the 6516 eligible participants, 366 (6%) were excluded due to insufficient data for frailty index score calculation (> 20% missing deficits [18]). A further 410 (6%) were excluded due to missing data on covariates.

### 2.2 Frailty Index

The frailty index is based on the proportion of accumulated deficits, such as symptoms, signs and laboratory abnormalities, which collectively contribute to an individual's age‐related vulnerability [1]. We selected deficits by following the five steps published in detail elsewhere [18]: (1) list variables measuring health problems—examples of these include disability, diseases and physical and cognitive impairments; (2) exclude variables with more than 15% values missing; (3) recode variables to reflect the severity of deficits on a scale from 0 (no deficit) to 1 (full expression of the deficit); (4) exclude variables that are too rare (< 1% with a full deficit) or too common (> 80% with a full deficit)—as these provide limited ability to distinguish between individuals in terms of ageing; and (5) screen all remaining variables for a positive association with age. In ISLAND, dichotomous variables were retained as‐is, while ordinal variables were transformed using proportional scoring methods based on the number of response categories or distribution percentiles. For instance, four‐level ordinal variables were coded as 0, 0.33, 0.67 and 1. Continuous variables were transformed into fractional scores based on predefined thresholds.

We calculated the ISLAND frailty index using the October 2020 data collection wave, which was selected due to being the first to contain complete and consistent data across all necessary instruments for frailty index construction. We used data from the Background and Health Survey, Dementia Risk Profile, Assessment of Quality of Life‐8 Dimensions, and Hospital Anxiety and Depression Scale [19]. Frailty index scores were computed as the proportion of deficits present among non‐missing variables and primarily used as a continuous variable in statistical analyses, although also categorised into three groups for descriptive purpose: low frailty (frailty index scores < 0.15), intermediate frailty (frailty index scores 0.15–0.25) and high frailty (frailty index scores > 0.25) [21]. The final composition of the frailty index is detailed in Table S1.

### 2.3 Remoteness

Participants were classified into residential remoteness categories (regional centres, rural towns and remote communities) based on the Modified Monash Model (MMM). The MMM is a classification system developed by the Australian Government that incorporates remoteness and town size to inform rural health workforce policy [22]. Corresponding MMM category codes (ranging from 0 to 7) were assigned based on participant postcode. Category 2 was classified as a regional centre, while small, medium and large rural towns (Categories 3 to 5) were grouped as rural towns. Remote and very remote communities (Categories 6 and 7) were combined as remote communities. Under the MMM, Tasmania, as a state, does not contain any areas classified as metropolitan (Category 1), with its most urbanised regions being regional centres.

### 2.4 Area‐Level Socioeconomic Status

Participants were classified into socioeconomic status groups using the Index of Relative Socio‐economic Advantage and Disadvantage (IRSAD), a composite measure developed by the Australian Bureau of Statistics [23]. The IRSAD summarises information about the economic and social conditions of people and households within an area. A low score indicates relatively greater disadvantage, while a high score reflects relatively greater advantage. Each participant was assigned an IRSAD score based on their residential postcode and categorised into low (more socioeconomic disadvantage), middle and high (more socioeconomic advantage) tertiles.

### 2.5 Covariates

In accordance with previous findings on frailty, age was considered as the key potential confounder in the relationship between remoteness and frailty [8]. Gender, educational attainment, migrant status and retirement status were also included in the descriptive analysis and were considered as covariates in the regression analyses [24]. Educational attainment was categorised as follows: ‘University qualification’—included bachelor's and higher degrees; ‘Post‐secondary’—included diplomas, associate degrees, certificates or apprenticeships; ‘School’—referred to individuals who completed high school; all other responses were grouped as ‘Other’. Migration status was categorised as non‐migrants or migrants to Australia, and further categorised as migrants from English‐speaking countries (ESC) and migrants from non‐English‐speaking countries (NESC).

### 2.6 Statistical Analysis

#### 2.6.1 Sample Characteristics

Descriptive statistics were used to summarise participant characteristics, including frailty index scores and frailty groups. Continuous variables were reported as mean (standard deviation) and medians (interquartile range), while categorical variables were reported as frequencies and percentages.

#### 2.6.2 Frailty Index Validation (Objective 1)

The validity of the ISLAND frailty index was assessed by comparing its statistical properties to published guidelines [18]. Frailty index scores should follow a gamma distribution, have a submaximal limit under 0.7, be positively associated with age, and be higher among women than men (reflecting the gender‐frailty paradox, where women live longer but accumulate more age‐related health deficits than men, on average [25]). We used multivariable linear regression models to determine the degree to which frailty index scores increased with advancing age and were higher among women than men after adjusting for age. For each criterion, scatter plots and histograms were used to visually confirm the distribution of frailty index scores.

#### 2.6.3 Regional and Sociodemographic Mapping (Objective 2)

To explore geographic and sociodemographic patterns of frailty, frailty index scores were mapped across age, gender, educational attainment, employment, migrant status and IRSAD. Frailty index scores were also presented across regions in Tasmania using geospatial analysis. Spatial variations were visualised using choropleth maps, and regional differences were displayed in box plots. Participants' residential postcodes were first linked to corresponding local government areas (LGA) codes using a postcode‐to‐LGA concordance [26]. Frailty index scores were then aggregated at the LGA level to calculate mean values for each region.

#### 2.6.4 Adjusted Regression Analysis (Objective 3)

Multiple linear regression was used to examine the relationship between MMM classifications and IRSAD groups with frailty index scores, in separate models, across three levels of statistical adjustment: (1) unadjusted; (2) adjusted for age; and (3) adjusted for age, gender, migrant status, educational attainment and retirement status. This stepwise approach allowed for a clearer understanding of how categories of covariates may influence associations. Model assumptions were checked using residual diagnostics, and multicollinearity was evaluated using variance inflation factors. To assess the robustness of findings, we conducted a sensitivity analysis by instead using a generalised linear model with a gamma distribution, which yielded comparable results.

Statistical analyses were undertaken using R (version 4.3.1), where *p* values below 0.05 were considered statistically significant. Data visualisation, including geospatial mapping and residual plots, was conducted using the ‘ggplot2’, ‘sf’, ‘scales’ and ‘patchwork’ packages.

## 3 Results

### 3.1 Descriptive Analysis

The final analytic sample consisted of 5740 participants, with a mean age of 69.3 years (SD = 8.0). Most participants were women (72%), held a university qualification (54%), were retired (59%) and resided in regional centres (69%). Men were older than women, on average (71.3 vs. 68.6 years), and a greater proportion of men were retired (66% of men vs. 56% of women). The most commonly reported ancestry was English or Irish (37%). Participants excluded due to missing data were slightly younger than the analytical sample, by 2.3 years, on average (Table S2).

### 3.2 Frailty Index Score (Objective 1)

**FIGURE 1:**
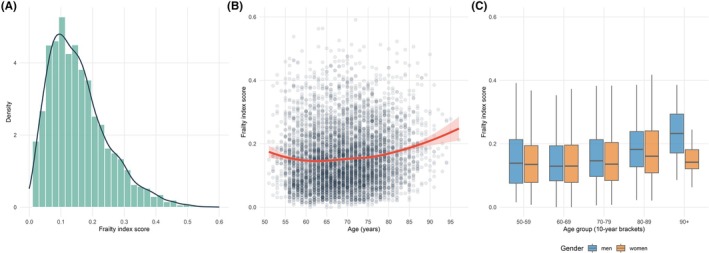
Frailty index scores by age and gender in ISLAND. (A) Histogram of frailty index scores (*n* = 5740) with overlaid density curve reflecting positively skewed gamma distribution. (B) Scatter plot showing the positive association between frailty index scores and age, with overlaid smoothing curve and 95% confidence band. (C) Box plot of frailty index scores by gender across age groups showing medians, interquartile ranges (IQR) and spread of non‐outlier values (1.5 × IQR). The theoretical range of the frailty index score is 0 to 1. ISLAND, Island Study Linking Ageing and Neurodegenerative Disease.

Frailty index scores followed a right‐skewed gamma distribution (Figure 1A), ranging from 0.000 to 0.591, with a mean of 0.16 (SD = 0.09). The density curve featured a sharp peak near zero, reflecting low deficit accumulation in most individuals, followed by a gradual decline towards higher scores. Most participants (55%) had low frailty (scores below 0.15), while a minority (15%) had high frailty (scores above 0.25). Frailty index scores tended to gradually increase with older age (Spearman's correlation = 0.19, *p* < 0.001) (Figure 1B). No statistically significant gender differences were observed in mean frailty index scores overall (men: 0.16 ± 0.09, vs. women: 0.15 ± 0.09, *p* = 0.51), with similarities present across most age groups (Figure 1C). In the 90+ years group, men had higher frailty index scores than women (mean frailty index score 0.24 vs. 0.16), although subgroup numbers were small (*n* = 20 men, 15 women). Lower frailty index scores were observed among younger age groups, participants who were employed, migrants from English‐speaking countries, people living in areas of high socioeconomic advantage, and those with a university qualification (Table 1).

**TABLE 1:** Frailty index scores across sociodemographic and geographic characteristics (*n* = 5740).

Characteristic	Low frailty *n* (%)	Intermediate frailty *n* (%)	High frailty *n* (%)	Frailty index score (mean ± SD, median [IQR])
Overall	3129 (55)	1726 (30)	885 (15)	0.16 ± 0.09, 0.14 [0.08, 0.21]
Age				
50–59	412 (58)	199 (28)	105 (15)	0.15 ± 0.10, 0.14 [0.08, 0.20]
60–69	1317 (58)	654 (29)	298 (13)	0.15 ± 0.09, 0.13 [0.08, 0.20]
70–79	1154 (54)	645 (30)	344 (16)	0.16 ± 0.09, 0.14 [0.09, 0.21]
80–89	234 (41)	215 (37)	128 (22)	0.18 ± 0.10, 0.17 [0.12, 0.24]
90+	12 (34)	13 (37)	10 (29)	0.20 ± 0.09, 0.18 [0.14, 0.26]
Gender				
Men	838 (51)	518 (32)	273 (17)	0.16 ± 0.09, 0.15 [0.09, 0.21]
Women	2291 (56)	1208 (29)	612 (15)	0.15 ± 0.09, 0.14 [0.08, 0.20]
Education				
University qualification	1800 (59)	903 (29)	370 (12)	0.14 ± 0.09, 0.13 [0.08, 0.19]
Post‐secondary	871 (50)	523 (30)	341 (20)	0.17 ± 0.10, 0.15 [0.09, 0.22]
School	392 (49)	259 (33)	146 (18)	0.17 ± 0.09, 0.15 [0.10, 0.22]
Other	66 (49)	41 (30)	28 (21)	0.17 ± 0.10, 0.15 [0.10, 0.23]
Employment status				
Employed	1408 (59)	657 (28)	309 (13)	0.15 ± 0.09, 0.13 [0.08, 0.19]
Retired	1721 (51)	1069 (32)	576 (17)	0.16 ± 0.10, 0.15 [0.09, 0.21]
Migrant status				
Non‐migrant	1469 (57)	755 (29)	344 (13)	0.15 ± 0.09, 0.13 [0.08, 0.20]
Migrant‐ESC	135 (59)	62 (27)	33 (14)	0.14 ± 0.09, 0.13 [0.08, 0.21]
Migrant‐NESC	1525 (52)	909 (31)	508 (17)	0.16 ± 0.10, 0.15 [0.09, 0.21]
MMM remoteness				
Regional centres	2218 (56)	1180 (30)	533 (14)	0.15 ± 0.09, 0.14 [0.08, 0.20]
Rural towns	835 (51)	503 (31)	311 (19)	0.16 ± 0.10, 0.15 [0.09, 0.22]
Remote communities	76 (48)	43 (27)	41 (26)	0.18 ± 0.11, 0.16 [0.09, 0.25]
IRSAD groups				
High IRSAD	1112 (60)	545 (29)	193 (10)	0.14 ± 0.08, 0.13 [0.08, 0.19]
Middle IRSAD	1064 (55)	578 (30)	302 (16)	0.16 ± 0.09, 0.14 [0.08, 0.21]
Low IRSAD	953 (49)	603 (31)	390 (20)	0.17 ± 0.10, 0.15 [0.09, 0.23]

*Note:* Low frailty, score < 0.15; intermediate frailty, score 0.15–0.25; high frailty, score > 0.25.

Abbreviations: ESC, English‐speaking countries; IQR, interquartile range; IRSAD, Index of Relative Socio‐economic Advantage and Disadvantage; MMM, Modified Monash Model; NESC, non‐English‐speaking countries; SD, standard deviation.

### 3.3 Regional and Sociodemographic Mapping (Objective 2)

**FIGURE 2:**
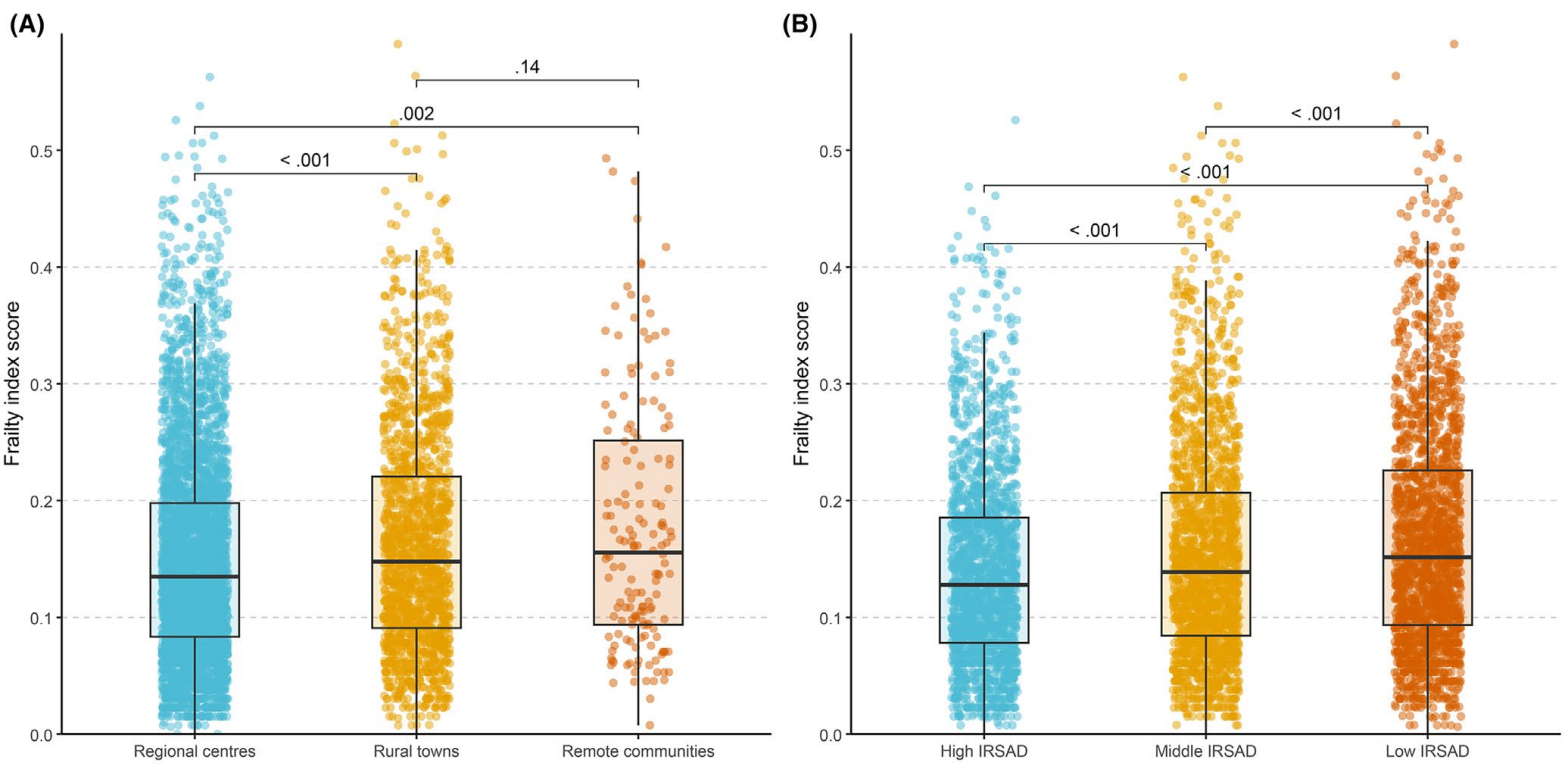
Distribution of frailty index scores by residential remoteness and IRSAD group. (A) Frailty index score by Modified Monash Model remoteness. (B) Frailty index score by IRSAD group. Box plots show frailty index scores with medians, interquartile ranges (IQR) and spread of non‐outlier values (1.5 × IQR). IRSAD, Index of Relative Socio‐economic Advantage and Disadvantage. The theoretical range of the frailty index score is 0 to 1.

**FIGURE 3:**
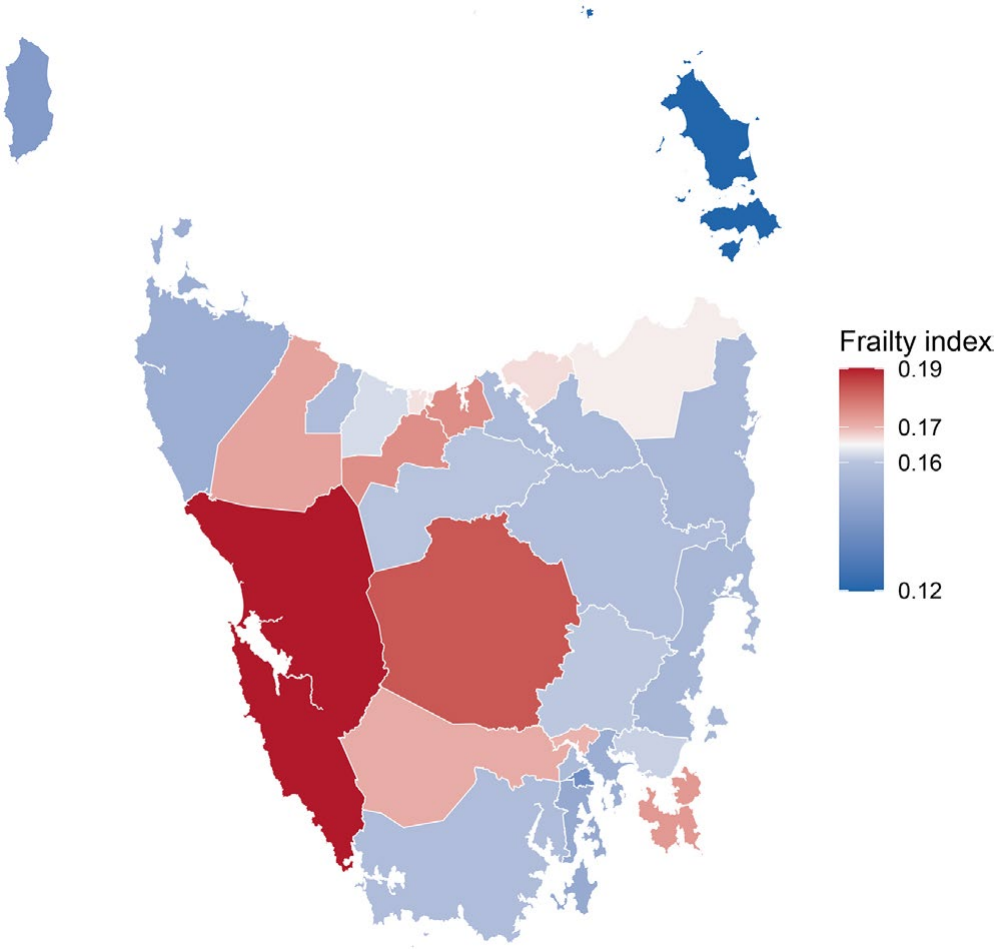
Mean frailty index scores across Tasmania by local government areas. Flinders Island (*n* = 10), King Island (*n* = 12) and Dorset (*n* = 42) had the smallest participant counts; all other regions had more than 50. The theoretical range of the frailty index score is 0 to 1.

Frailty index scores differed significantly across residential remoteness categories (Figure 2A). Participants in rural towns (mean FI: 0.16 ± 0.10) and remote communities (0.18 ± 0.11) had significantly higher mean frailty index scores than those in regional centres (0.15 ± 0.09). No statistically significant difference was observed between rural towns and remote communities. The proportion of participants classified as having high frailty increased with remoteness: 14% in regional centres, 19% in rural towns and 26% in remote communities. Geospatial mapping revealed clusters of elevated frailty index scores in non‐capital regions in central and western regions of Tasmania (Figure 3).

Frailty index scores also varied by IRSAD (Figure 2B). The low IRSAD group (the most disadvantaged areas) had higher mean frailty index scores (0.17 ± 0.10) than those from middle (0.16 ± 0.09) and high (0.14 ± 0.08) IRSAD groups (the most socioeconomically advantaged areas). The proportion of individuals with high frailty increased steadily with socioeconomic disadvantage: 10% in high, 16% in middle and 20% in low IRSAD groups.

### 3.4 Regression Outputs (Objective 3)

**FIGURE 4:**
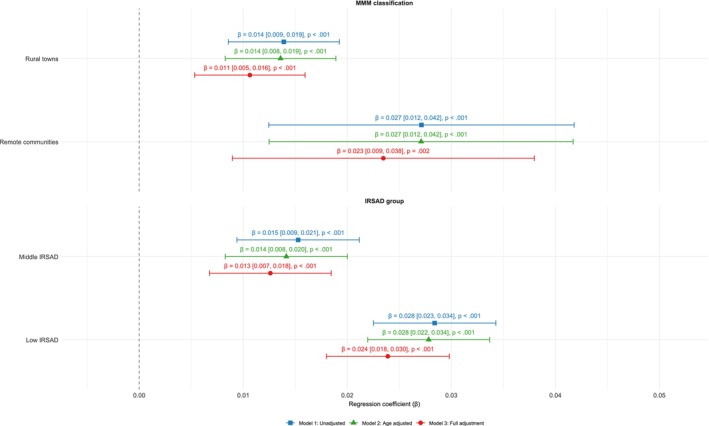
Association between residential remoteness and socioeconomic status with frailty index scores. Coefficients (β) and 95% confidence intervals were derived from multiple linear regression models at different levels of covariate adjustment. Reference categories were regional centres and high IRSAD (Index of Relative Socio‐economic Advantage and Disadvantage). Model 3 was adjusted for age, gender, educational attainment, migrant status and retirement status.

Linear regression analyses supported an association between both remoteness and IRSAD with frailty (Figure 4). In the unadjusted model, compared with participants living in regional centres, frailty index scores were higher among those living in rural towns and those living in remote communities. A similar gradient was observed for IRSAD socioeconomic groups. Associations remained mostly unchanged after adjusting for age, suggesting that age differences between remoteness and IRSAD groups accounted for only a small proportion of frailty disparities. Compared with unadjusted associations, following full covariate adjustment, the strength of the estimate associated with rural towns decreased by 21% from 0.014 to 0.011, while the estimate associated with remote communities decreased by 15% from 0.027 to 0.023. For IRSAD, compared with the high group (the most socioeconomically advantaged areas), the estimate associated with the middle group decreased by 13% (from 0.015 to 0.013), while the estimate associated with the low group (the most socioeconomically disadvantaged areas) decreased by 14% (from 0.028 to 0.024). Despite attenuation, all associations remained statistically significant, suggesting that remoteness and socioeconomic status contributed to frailty disparities beyond the measured covariates.

## 4 Discussion

This study developed and validated a frailty index for participants of the ISLAND cohort in Tasmania, Australia, mapping its distribution across geographic and sociodemographic strata while exploring regional disparities in frailty burden. Among the ISLAND web‐based cohort, we found that frailty index scores were elevated in both rural and remote communities, and also in socioeconomically disadvantaged areas, even after adjusting for key sociodemographic determinants such as age, gender and educational attainment. Our findings provide perspectives on how ageing, regionality and sociodemographic determinants intersect to influence health inequities in Tasmania's population and beyond. In the light of the strong associations between frailty and later disability and dementia [27], these findings reinforce the potential value of incorporating frailty measurement into healthcare resource allocation and planning.

The ISLAND frailty index scores followed a gamma distribution, had a submaximal limit under 0.7 and increased with older age, aligning with published guidelines [18]. We noted a lack of gender differences, contrasting with earlier work establishing higher degrees of frailty in women than men at any chronological age [25, 28]. This discrepancy suggests selective participation bias may exist where healthier women enrolling in the study could attenuate gender disparities. Another possible explanation is that some health conditions that are more common in women were not explicitly captured as standalone items in ISLAND and therefore not considered in the frailty index. For example, conditions such as osteoporosis and arthritis are more prevalent in older women and have a strong impact on physical function and frailty [29], but were not available in ISLAND. At the same time, some conditions with higher prevalences in men, such as heart disease [30] and hearing loss [31], were available. While higher frailty scores among women are commonly reported, changes in gender roles, increased healthcare access for women [32], and uptake of medical interventions may be narrowing these gaps.

In Australia, there has been variability in findings with regard to the geographical distribution of frailty [33, 34]. Here, we found geographic disparities in frailty that remained after adjustment for age, gender, educational attainment, migrant status and retirement. This suggests that remoteness may represent an independent risk factor for frailty, with participants in rural and remote communities having higher frailty index scores than their regional counterparts. According to our fully adjusted regression model results, individuals residing in remote communities had frailty index scores that were 0.03 points higher than those living in regional centres—equivalent to approximately one additional health deficit. A difference of this magnitude is clinically meaningful, as a 0.03 change in the frailty index can serve as a benchmark for evaluating the impact of interventions and significant health changes over time [35]. From a population health perspective, even modest increases in frailty index scores signify a heightened risk of adverse outcomes [36, 37], which may translate into substantial disparities in health service demand [38], particularly in remote settings with constrained access to care. We also found similar patterns for socioeconomic disadvantage, with a clear area‐level socioeconomic gradient where mean frailty index scores increased progressively from low to high IRSAD groups. These findings support previous work showing people living in more deprived neighbourhoods or with lower wealth consistently have higher rates and severity of frailty, even after accounting for age, gender and health behaviours [33].

The design and implementation of effective place‐based interventions to reduce the observed regional disparities in frailty rely upon an understanding of the mechanisms underlying these relationships. Reduced access to healthcare services and preventive care, limited transportation options, and scarcity of specialist care in remote areas can delay detection and management of chronic conditions that ultimately contribute to frailty [39]. Mobile health clinics and supported telehealth services could potentially bridge the geographic gap, facilitating earlier diagnosis and consistent chronic disease management in underserved regions [40, 41]. Furthermore, management strategies require adaptation to the local service landscape to ensure care plans remain feasible where specialist support is limited [40, 41]. Additionally, environmental and occupational factors such as higher exposure to physical labour, injury risk or harsh climatic conditions may also exacerbate frailty in remote and disadvantaged socioeconomic settings [4]. These stressors may be mitigated by establishing protective social and physical environments, such as community gardens, or by implementing home‐based functional training programmes to build resilience against injury and provide safer contexts for physical activity [40, 41]. Addressing these structural disparities requires an integrated and targeted approach to frailty prevention that extends beyond individual‐level interventions [42].

It is feasible to construct an electronic, automated frailty index from routinely collected health data to enable systematic frailty screening and monitoring. Electronic frailty indices have been developed and validated using electronic patient records in several countries, including the United Kingdom, China, Spain and Australia [1, 33, 43, 44, 45], and have utility in predicting adverse outcomes, such as mortality. Embedding this approach in primary care represents a cost‐effective and sustainable approach, enabling ongoing monitoring as part of routine patient care [45]. In Italy, an electronic frailty index derived from routinely collected primary‐care records showed independent associations with hospitalisation, demonstrating its value in guiding healthcare resource allocation [46]. The use of an electronic frailty index optimises patient care for frail, older individuals, but may also prove useful in enabling the population monitoring of frailty and the evaluation of interventions aimed at reducing regional disparities in frailty.

Strengths of this study include rigorous frailty index construction and validation adhering to the 10‐step construction framework by Theou et al. [18]. By integrating self‐reported disease, functional and psychosocial deficits, the ISLAND frailty index provides a multidimensional measure of health vulnerability [47]. This integration is consistent with contemporary approaches to multidimensional frailty assessment in ageing research [48], which acknowledge non‐physical domains such as psychological distress that can influence frailty progression [47]. Other conceptualisations of frailty exist too, such as physical frailty [6] and social frailty [49], which are strongly associated with adverse outcomes. However, findings are constrained by the following limitations. First, there is selection bias from web‐based recruitment, which likely under sampled frail subgroups such as people using residential aged care. Second, the cross‐sectional design precluded causal inference, but the ISLAND cohort's ongoing longitudinal design will allow future investigations into frailty trajectories and their interaction with other adverse health outcomes. Third, reliance on self‐reported deficits in frailty index construction (e.g., functional limitations, chronic conditions) may introduce recall bias. Objective measures (e.g., gait speed, grip strength) could strengthen future iterations. These measures are available for a subset of participants who attend the ISLAND Clinc [50, 51]. Integrating clinical assessments with cohort data may improve the precision and validity of frailty estimation in future work. Fourth, frailty calculations were based on data collected approximately 5 years ago, and population health profiles may have changed over time. Fifth, participants excluded due to missing data were slightly younger than the analytical sample (Table S2). While the absolute age difference was small, this may limit generalisability to younger age groups. Finally, unmeasured confounders, such as income, transportation access and healthcare use, further limit interpretability. While this study focussed on remoteness and area‐level socioeconomic disadvantage separately, these factors likely intersect with one another and with individual‐level characteristics such as social engagement and other lifestyle factors. Future research is needed to systematically evaluate how these protective health behaviours and psychosocial factors interact with regionality to influence frailty trajectories.

## 5 Conclusions

The findings from this study suggest that incorporating frailty assessments into regional public health planning is a viable strategy to identify vulnerabilities of ageing populations. Clinically, these disparities highlight the value of opportunistic frailty screening during routine encounters to trigger early, tailored interventions that account for local service barriers. Given the higher frailty scores observed in rural and remote communities, as well as in socioeconomically disadvantaged areas, targeted policies and services are needed to support ageing populations outside of urban centres. Interventions should prioritise enhancing healthcare access through mobile and telehealth services while bolstering community resilience through social and physical activity programmes. Addressing regional disparities is fundamental to equitable ageing.

## Funding

The ISLAND Project was supported by the Australian Government's Medical Research Future Fund, the National Health and Medical Research Council (2004051), the J.O. and J.R. Wicking Trust (Equity Trustees), St. Luke's Health and the Masonic Centenary Medical Research Foundation. This project was supported by a National Health and Medical Research Council (NHMRC) Centre of Research Excellence grant (ID: 2015821). ZL was supported by an Australian Government Research Training Program (RTP) Scholarship.

## Ethics Statement

The data used in this article were derived from the Island Study Linking Ageing and Neurodegenerative Disease (ISLAND) project. The ISLAND project was approved by the University of Tasmania Human Research Ethics Committee (HREC; reference 18264).

## Conflicts of Interest

The authors declare no conflicts of interest.

## Supporting information


**Table S1:** Composition of the frailty index.

**Table S2:** Characteristics of analytical sample vs. excluded sample.

## Data Availability

The data that support the findings of this study are available from the corresponding author upon reasonable request.
