# Supplementary material for: Regional and Socioeconomic Disparities in Frailty Across Tasmania: Evidence From Island Study Linking Ageing and Neurodegenerative Disease
Source: Australas J Ageing. 2026 Mar 9;45(1):e70144. doi: 10.1111/ajag.70144 (PMC12972236; doi:10.1111/ajag.70144)
Supplement: Supplementary file 1 — Table S1: Composition of the frailty index. [file AJAG-45-0-s001.docx]

**Supplementary Table 1:** Composition of the frailty index

| **No.** | **Item** | **Description** | **Scoring** |
| --- | --- | --- | --- |
| 1 | Medications | Are you currently taking any prescription medications or hormonal supplements? | 0 = no, 1 = yes |
| 2 | Depression | Assessed by Hospital Anxiety and Depression Scale | 0 = normal, 1 = abnormal (11-21) / borderline abnormal (8-10) |
| 3 | Anxiety | Assessed by Hospital Anxiety and Depression Scale | 0 = normal, 1 = abnormal (11-21) / borderline abnormal (8-10) |
| 4 | Abnormal weight | BMI over 30 as obesity, under 18.5 as underweight | 0 = BMI between 18.5 to 30, 1 = BMI > 30 or < 18.5 |
| 5 | Low walking time | For each day of the week, how many minutes do you typically spend walking at a time? | 0 = walk 30 mins or more per day, 1 = walk less than 30 mins per day |
| 6 | Low physical activity | For each day of the week, how many minutes do you typically spend doing moderate physical activities like carrying light loads, bicycling at a regular pace, dancing, water aerobics, or doubles tennis? | 0 = exercise 20 mins or more per day, 1 = exercise less than 20 mins per day |
| 7 | Diabetes | Have you been told by a doctor or other health professional that you have diabetes or pre-diabetes (or high sugar levels)? | 0 = no, 1 = yes |
| 8 | High cholesterol level | Have you been told by a doctor or other health professional that you have high total cholesterol levels? | 0 = no, 1 = yes |
| 9 | High blood pressure | Have you been told by a doctor or other health professional that you have high blood pressure or hypertension? | 0 = no, 1 = yes |
| 10 | Legally blind | Are you legally blind? | 0 = no, 1 = yes |
| 11 | Psychiatric disease | Have you been diagnosed with a mood disorder or any other mental health condition? | 0 = no, 1 = yes |
| 12 | Memory impairment | have you been told by a doctor that you have a memory impairment? | 0 = no, 1 = yes |
| 13 | Kidney disease | Have you been diagnosed with kidney disease? | 0 = no, 1 = yes |
| 14 | Heart disease | Have you been diagnosed with heart disease? | 0 = no, 1 = yes |
| 15 | Hearing impairment | Do you have a hearing impairment | 0 = no, 1 = yes |
| 16 | Head injury | Have you ever had a serious head injury? | 0 = no, 1 = yes |
| 17 | Epilepsy | Have you been diagnosed with epilepsy | 0 = no, 1 = yes |
| 18 | Dementia diagnosis | Have you been told by a doctor that you have dementia? | 0 = no, 1 = yes |
| 19 | CNS disease | Have you been diagnosed with a central nerve system degenerative disease? | 0 = no, 1 = yes |
| 20 | Cancer | Have you been diagnosed with cancer? | 0 = no, 1 = yes |
| 21 | Vitamin B12 deficiency | have you been diagnosed with vitamin B12 deficiency? | 0 = no, 1 = yes |
| 22 | Serious pain | How often do you experience serious pain? | 0 = very rarely, 0.25 = less than once a week, 0.5 = once or twice a week, 0.75 = three to four times a week, 1 = most of the time. |
| 23 | Hearing quality | How well can you hear (using your hearing aid if needed)? | 0 = I have excellent hearing, 0.2 = I hear normally, 0.4 = I have some difficulty hearing, or I do not hear clearly (e.g. when there is background noise), 0.6 = I have difficulty hearing things clearly. Often I do not understand what is said. I usually do not take part in conversations because I cannot hear what is said. 0.8 = I hear very little, 1 = I am completely deaf. |
| 24 | Help with tasks | How much help do you need with jobs around your place of residence (e.g. preparing food, cleaning, gardening)? | 0 = I can do all these tasks very easily without any help, 0.25 = I can do these tasks relatively easily without help, 0.5 = I can do these tasks only very slowly without help, 0.75 = I cannot do most of these tasks unless I have help, 1 = I can do none of these tasks by myself. |
| 25 | Difficulty getting around | How easy or difficult is it for you to get around by yourself outside your place of residence (e.g. to go shopping, visiting)? | 0 = getting around is enjoyable and easy, 0.2 = I have no difficulty getting around outside my place of residence, 0.4 = a little difficulty, 0.6 = moderate difficulty, 0.8 = a lot of difficulty, 1 = I cannot get around unless somebody is there to help me. |
| 26 | Vison quality | How well can you see (using your glasses or contact lenses if they are needed)? | 0 =I have excellent sight, 0.2 = I see normally, 0.4 = I have some difficulty seeing things sharply. (e.g. small print, objects in the distance, or watching television), 0.6 = I have a lot of difficulty seeing sharply, 0.8 = I only see general shapes, 1 = I am completely blind |
| 27 | Pain interference | How often does pain interfere with your usual activities? | 0 = none at all, 0.33 = I have moderate pain, 0.66 = I suffer from severe pain, 1 = I suffer unbearable pain. |
| 28 | Self-care difficulty | How difficult is it for you to wash, toilet, dress yourself, eat or care for your appearance? | 0 = these things are very easy for me to do, 0.25 = I have no real difficulty in doing these things, 0.5 = I find some of these things difficult, but I manage to do them on my own, 0.75 = many of these things are difficult, and I need help to do them, 1= I cannot do these things by myself at all. |
| 29 | Enthusiasm | How enthusiastic do you feel? | 0 = extremely, 0.25 = very, 0.5 = somewhat, 0.75 = not much, 1 = not at all |
| 30 | Ease of movement | How easy or difficult is it for you to move around (using any aids or equipment you need e.g. a wheelchair, frame or stick)? | 0 = I am very mobile, 0.2 = I have no difficulty with mobility, 0.4 = I have some difficulty with mobility (for example, going uphill), 0.6 = I have difficulty with mobility. I can go short distances only, 0.8 = I have a lot of difficulty with mobility. I need someone to help me, 1 = I am bedridden. |
| 31 | Sleep trouble | How often do you have trouble sleeping? | 0 = never, 0.25 = almost never, 0.5 = sometimes, 0.75 = often, 1 = all the time |
| 32 | Communication difficulty | How well do you communicate with others (talking, signing, texting, being understood by others and understanding them)? | 0 = I have no trouble being understood, 0.33 = I have some difficulty being understood by people who do not know me, 0.66 = I am understood only by people who know me, 1 = I cannot adequately communicate with others. |
| 33 | Energy level | Thinking about how much energy you have to do the things you want to do? | 0 = always full of energy, 0.25 = usually full of energy, 0.5 = occasionally energetic, 0.75 = usually tired and lacking energy, 1 = always tired and lacking energy. |
