# Supplementary material for: Regional and Socioeconomic Disparities in Frailty Across Tasmania: Evidence From Island Study Linking Ageing and Neurodegenerative Disease
Source: Australas J Ageing. 2026 Mar 9;45(1):e70144. doi: 10.1111/ajag.70144 (PMC12972236; doi:10.1111/ajag.70144)
Supplement: Supplementary file 2 — Table S2: Characteristics of analytical sample vs. excluded sample. [file AJAG-45-0-s002.docx]

**Supplementary Table 2:** Characteristics of analytical sample vs. excluded sample

| **Characteristic** | **Analytical Sample (n = 5,740)** | **Excluded Sample (n = 776)** | ***p*-value** |
| --- | --- | --- | --- |
| Age, years |  |  | < 0.001 |
| Mean ± SD | 69.3 ± 8.02 | 67.0 ± 8.16 |  |
| Gender, n (%) |  |  | 0.50 |
| Men | 1629 (28) | 223 (29) |  |
| Women | 4111 (72) | 553 (71) |  |
